# Supplementary material for: Resolving the full spectrum of human genome variation using Linked-Reads
Source: Genome Res. 2019 Apr;29(4):635–45. doi: 10.1101/gr.234443.118 (PMC6442396; doi:10.1101/gr.234443.118)
Supplement: Supplemental Material [file supp_gr.234443.118_Supplemental_longranger-2.2.zip › longranger-2.2.tar.bz2!/lr2.2-public/lib/stan/cmdstan/stan/doc/api/html/structstan_1_1gm_1_1program-members.html]

Stan: Member List


|  |  |
| --- | --- |
|  | Stan  2.5.0  probability, sampling & optimization |


- Main Page
- Related Pages
- Namespaces
- Classes
- Files

- Class List
- Class Index
- Class Hierarchy
- Class Members

All Classes Namespaces Files Functions Variables Typedefs Enumerations Enumerator Friends Macros Pages

- stan
- gm
- program

stan::gm::program Member List

This is the complete list of members for stan::gm::program, including all inherited members.

|  |  |  |
| --- | --- | --- |
| data\_decl\_ | stan::gm::program |  |
| derived\_data\_decl\_ | stan::gm::program |  |
| derived\_decl\_ | stan::gm::program |  |
| function\_decl\_defs\_ | stan::gm::program |  |
| generated\_decl\_ | stan::gm::program |  |
| parameter\_decl\_ | stan::gm::program |  |
| program() | stan::gm::program |  |
| program(const std::vector< function\_decl\_def > &function\_decl\_defs, const std::vector< var\_decl > &data\_decl, const std::pair< std::vector< var\_decl >, std::vector< statement > > &derived\_data\_decl, const std::vector< var\_decl > &parameter\_decl, const std::pair< std::vector< var\_decl >, std::vector< statement > > &derived\_decl, const statement &st, const std::pair< std::vector< var\_decl >, std::vector< statement > > &generated\_decl) | stan::gm::program |  |
| statement\_ | stan::gm::program |  |


---

[ Stan Home Page ]

*© 2011–2014,
Stan Development Team.*
